# Supplementary material for: Two-Sex Life Table Analysis for Optimizing Beauveria bassiana Application against Spodoptera exigua (Hübner) (Lepidoptera: Noctuidae)
Source: J Fungi (Basel). 2024 Jul 4;10(7):469. doi: 10.3390/jof10070469 (PMC11277660; doi:10.3390/jof10070469)
Supplement: Supplementary file 1 [file jof-10-00469-s001.zip › jof-3026625-supplementary.pdf]

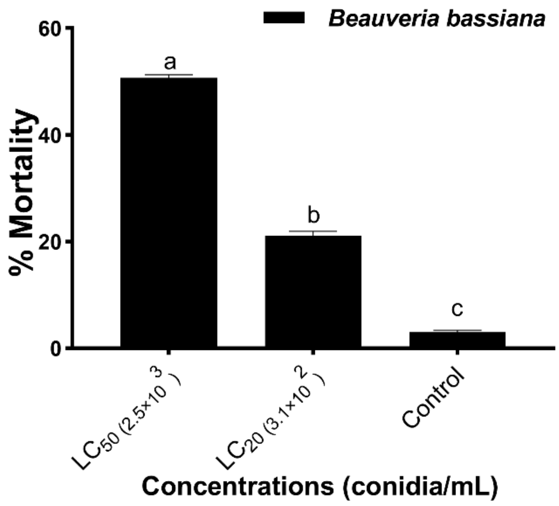

Figure S1. Experimental validation of lethal (LC_{50}) and sublethal (LC_{20}) concentrations of *B. bassiana* on *S. exigua.*
